# Limited-Distance Pollen Dispersal and Low Paternal Diversity in a Bird-Pollinated Self-Incompatible Tree

**DOI:** 10.3389/fpls.2022.806217

**Published:** 2022-02-25

**Authors:** Wen-Qian Xiang, Pastor L. Malabrigo, Liang Tang, Ming-Xun Ren

**Affiliations:** ^1^Key Laboratory of Ministry of Education for Genetics and Germplasm Innovation of Tropical Special Trees and Ornamental Plants, Hainan University, Haikou, China; ^2^Center for Terrestrial Biodiversity of the South China Sea, Hainan University, Haikou, China; ^3^Department of Forest Biological Sciences, College of Forestry and Natural Resources, University of the Philippines Los Baños, Los Baños, Philippines

**Keywords:** mating system, paternity analysis, pollen flow, microsatellite DNA, genetic diversity, Bombacoideae

## Abstract

Bird pollination in Asia is regarded as an uncommon phenomenon and, therefore, only a few investigations on mating pattern and paternity in fruits of Asian bird-pollinated plants have been conducted. Here, we examined spatial genetic structure, pollen dispersal, and multiple paternity in a natural population of *Bombax ceiba* (*B. ceiba*) (Malvaceae) in Hainan Island, South China, using simple sequence repeat (SSR) markers. A low genetic diversity (*H*_*e*_ = 0.351 ± 0.0341 and 0.389 ± 0.043, respectively, for adults and offspring) and bottleneck effects were observed. Genetic kinship was significant within 400 m or in 1,800–3,800 m. Both the mating pattern and paternity analysis confirmed obligate xenogamy and a low multiple paternity in *B. ceiba*. There was a strongly negative relationship between the frequency of matings and the distance between mating pairs. The average pollen dispersal distance was 202.89 ± 41.01 m (mean ± SE) and the farthest distance of > 1 km was recorded. Realized mating events showed an extremely leptokurtic distribution within 1,200 m, suggesting that the pollen dispersal distance was consistent with the optimal foraging theory of generalist birds such as *Zosterops* spp. and *Pycnonotus* spp. Paternity per tree ranged from two to six and the average effective number of pollen donors per maternal plant was 3.773, suggesting a low level of paternity diversity as compared to other bird-pollinated plants. We concluded that optimal foraging behavior by generalist birds could explain the leptokurtic pollen dispersal distribution and predominantly near-neighbor matings in *B. ceiba*. The limited pollen dispersal distance and low multiple paternity were consistent with low fruit setting rate (3.27 ± 0.93%) in this self-incompatible tree, which was caused mainly by the restricted flight distance of birds and human disturbances. Low genetic diversity and significant spatial genetic structure might have largely resulted from logging and human collection of fruits.

## Introduction

Birds are one of the most diverse group of ecosystem service providers. More than 900 bird species pollinate members of ca. 500 of the 13,500 vascular plant genera, making bird pollination an essential component of the ecosystem (Sekercioglu, 2006). This pollination function that birds provide is mainly an outcome of their foraging behavior. Birds act as mobile links that transfer pollen within and between plant populations (Bezemer et al., 2016). Often times, birds could be more effective pollinators than insects such as honeybees because their greater potential mobility (Southerton et al., 2004; Coimbra et al., 2020). In Myrtaceae, Proteaceae, Polemoniaceae, and other bird-pollinated species, the pollen dispersal distance within the population is usually within 200 m (Krauss et al., 2009; Bezemer et al., 2016). At larger scales, birds that undertake long distance movements in pursuit of patchy nectar supplies are more capable of mediating pollen dispersal distances of several kilometers when populations are fragmented (Byrne et al., 2007). Further, high energy needs may promote birds to visit several plants on a single foraging bout, which in combination with their ability to travel long distances help in promoting xenogamous pollination. Moreover, pollination by highly mobile and long-distance flying birds can reduce the risk of genetic erosion associated with demographic bottlenecks and genetic drifts (Bezemer et al., 2016) and also increases the resilience of naturally fragmented and genetically insular plant populations by buffering genetic erosion (Breed et al., 2015).

Bird pollination research has largely focused on Scrophulariaceae, Labiatae, Costaceae, and Proteaceae families of plants in South America, Africa, and Australia (Krauss et al., 2017). This may be attributed to the highest diversity of effectively specialized nectarivorous birds in tropics and in the Southern Hemisphere, which include sunbirds (African and Asian tropics), honeyeaters, lorikeets (eastern Australia and New Guinea), and hummingbirds (the Andes and central American mountains). For a long time, bird pollination systems were generally overlooked in Asia because sunbirds (Nectariniidae) are the only specialized nectar-feeding birds (Krauss et al., 2017) and only few plant species are pollinated by specialist nectar-feeding birds. However, increasing number of studies for almost two decades show that generalist birds act as alternative pollinators in Asia (Roguz et al., 2018). Remarkably, generalist passerines have been demonstrated as the most effective pollinators for winter flowering plants in regions where sunbirds are absent, especially in dry seasons and in the islands (Chen et al., 2019). These include species such as *Fritillaria imperialis* (Roguz et al., 2021), *Firmiana* spp. (Huang et al., 2018), *Rhodoleia* spp. (Gu et al., 2010), *Woodfordia* spp. (Raju, 2005), Rhododendraceae (Song et al., 2019), and Bombacoideae (Xiang and Ren, 2019).

Previous studies have mostly focused on observing pollination behaviors or analyzing the reproductive ecology of tree species pollinated by generalist birds (Huang et al., 2018). It is conspicuous that such a pollination system is relatively common in Asia and on tropical islands. Yet, questions related to mating behavior, pollen dispersal, and genetic structure of plant species pollinated by generalist birds remain unexplored. Under extreme environmental conditions such as dry weather (Stiles, 1978), on island habitats (Chen et al., 2019), and due to increasing human interference (Byrne et al., 2007), a better understanding of the mating system of generalist-pollinated species and of how birds influence mating patterns is further warranted. Identifying the underlying behavioral mechanisms of bird pollinators responsible for pollen movement would deepen the understanding of evolutionary and ecological consequences of bird pollination for plant mating.

**FIGURE 1:**
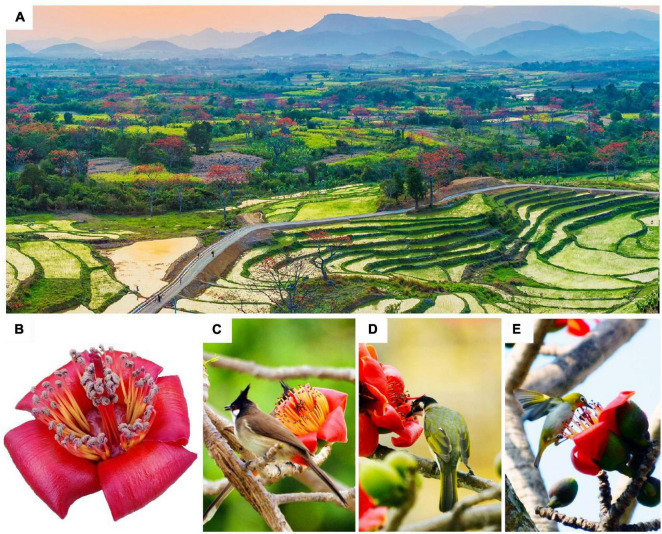
*Bombax ceiba* (*B. ceiba*) in Hainan Island. **(A)** A flowering population in west Hainan Island. **(B)** A representative picture of floral syndromes. Note that stamens are in two whorls. *B. ceiba* flowers were mainly visited by *Pycnonotus jocosus* **(C)**, *Brachypodius atriceps*, **(D)** and *Zosterops palpebrosus* **(E)**.

The tropical tall tree species of *Bombax ceiba* (*B. ceiba*) L. (Bombacoideae, Malvaceae) is an umbrella species in dry and hot forest areas of Asia because they can provide food, shelter, roosting, and breeding sites to a large number of birds as well as insects and form the major structural and functional basis of forest ecosystems (Jain et al., 2011). It is found in temperate and tropical regions of Asia such as India, China-Indochina Peninsula, Indonesia, Malaysia, Philippines, Sri Lanka, and Northern Australia. *B. ceiba* is large deciduous trees, distributed mainly in arid-hot valleys, roadsides, and open areas in tropical and subtropical Asia (Barwick, 2004; Zhou et al., 2015). In southern China, it is a significant economic tree species and a tourist resource, which is often disturbed by urbanization, felling, and other human activities. *B. ceiba* is a typical bird-pollinated plant with big flowers that have bright upward corolla and copious dilute nectar (Raju et al., 2005). The flowering of *B. ceiba* peaks during late February and early March, which is the dry season in southern China, especially in Hainan Island (data from National Meteorological Information Center).^1^ At this time, there are only limited number of plants that are flowering in southern China and the copious dilute nectar of *B. ceiba* acts as the main food and a safe water resource for generalist birds. Li et al. (2016) and Xiang and Ren (2019) confirmed that migratory omnivorous (generalist) birds, such as those of Zosteropidae, Sturnidae, and Laniidae families, were the frequent and efficient pollinators of *B. ceiba* during the peak flowering in China, especially in Hainan Island and dry-hot valleys of Yunnan (Figure 1 and Table 1).

**TABLE 1:** The different types of birds that visit *Bombax ceiba* as well as their frequency of visits, habits, and flight capability.

Species	Group members	Visit frequency (Visits*tree^–1^*h^–1^)	Long distance flight capability (> 5 km)	Home range impacts on axial variance	References
*Zosterops palpebrosus*	10–15	4.98 ± 0.72	Yes	Typically < 3 ha	Zhao, 2001; Higgins et al., 2006
*Pycnonotus xanthorrhous*	3–5	0.47 ± 014	No	No data	Zhao, 2001
*Pycnonotus jocosus*	3–10	0.41 ± 0.15	No	No data	Zhao, 2001
*Acridotheres cristatellus*	2–4	0.20 ± 0.07	Yes	No data	Zhao, 2001
*Sturnus sinensis*	2–6	0.08 ± 0.04	Yes	Typically < 3 ha	Zhao, 2001; Higgins et al., 2006
*Lanius* sp.	1–2	0.67 ± 0.16	Yes	Typically < 3 ha	Zhao, 2001; Higgins and Peter, 2002
*Parus* sp.	3–5	0.23 ± 0.08	No	No data	Zhao, 2001

In this study, we analyzed genetic diversity, mating system, gene flow, and multiple paternity of *B. ceiba* that are pollinated by generalist birds in the Hainan Island of China. Specifically, this study addresses the following two questions: (i) What is the mating system and pollen dispersal of *B. ceiba*? and (ii) How do generalist birds affect genetic diversity and mating patterns? We hypothesize that: (i) limited-distance pollen dispersal and low paternal diversity may be due to high density of *B. ceiba* individuals and bird habits such as the restricted flight distance of birds and optimum foraging behavior of birds and (ii) pollination by generalist birds may result in high outcrossing rate, but low genetic diversity in *B. ceiba* due to human disturbances.

## Materials and Methods

### Study Site

**FIGURE 2:**
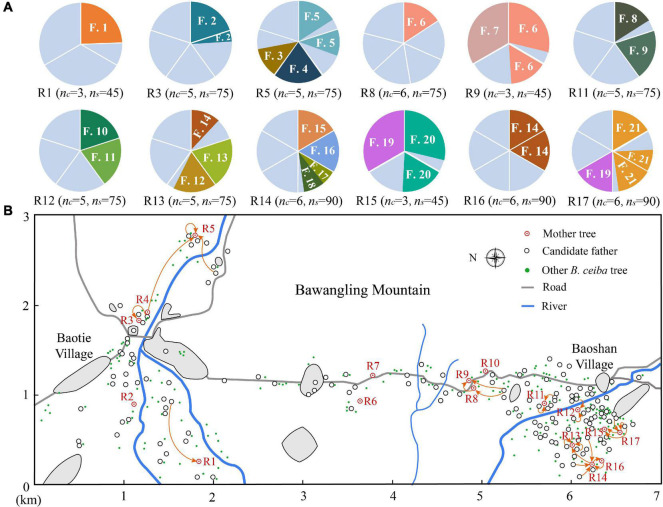
Pollen flow and multiple paternity of *B. ceiba*. **(A)** Composition of fathers in individual trees. *n*_*c*_, number of capsules analyzed in each tree; *n*_*s*_, number of seeds analyzed in each tree; F, seedlings assigned father; different colors in the pie chart represent seedlings assigned to different fathers and gray color indicates seedlings not assigned to a pollen parent within the study population. **(B)** Pollen flow of *B. ceiba* individuals in the study stand. The gray area represents buildings.

This study was conducted on a wild population of *B. ceiba* trees that were located at the forest margins near the Bawangling National Nature Reserve, west Hainan Island (18°53′–19°30′N, 108°38′–109°17′E; Figures 1, 2). *B. ceiba* is a common species in this region and this study site contains the most concentrated areas of *B. ceiba* in Hainan Island. Studied population is located across Baotie to Baoshan villages, which are connected by a rural road and are surrounded by *Hevea brasiliensis*. The total area of this study site is 2,100 hectares (Figure 2) and the *B. ceiba* trees were distributed on the paddy fields and sugarcane fields. The area is dominated by a monsoon climate, marked with a distinct wet (May to October) and a dry season (November to next April), and witnesses the lowest annual precipitation (about 1,700 mm) on Hainan Island. The annual average temperature is about 24.3°C (data from Meteorological Service of Hainan).^2^

### Study Species

The flowering season of *B. ceiba* is from early February to late March and the life span of one single flower is 7–12 days. At midnight, hundreds of the mature buds begin to open. *B. ceiba* is a protandry plant and the anthers dehisce almost immediately after the flowers open and pollen activity was about 80%. After anther dehiscence, the stigma becomes receptive and the function between the sexes overlapped. The staminate phase can last for about 30 h, while the pistillate phase of *B. ceiba* can last around 3–4 days.

The capsule maturation period of *B. ceiba* in Hainan Island is between March and May. Capsules are ellipsoid and dehisce after the ripening. Many seeds are produced (about 200), which are obovate, smooth, and fibrous from outside and form fluffy spheres of seed coats. The seeds are located in the center of the sphere and are dispersed far away by the wind.

### Bird Observation and Hand-Pollination Treatments

On around 10 consecutive sunny days, between 06:00 and 18:00 h in February 2018, the number and type of bird visits per day in the 10 blooming individuals were recorded and photographed everyday in studied population.

Hand-pollination experiments were conducted in the wild population to estimate mating systems of *B. ceiba*. Five pollination treatments were applied on different branches on four trees (Table 2): (1) Bagged only (*n* = 20 flowers), bagging mature buds without any hand pollination; (2) emasculation (*n* = 20 flowers), bagging mature buds and all the anthers were cutoff; (3) autogamy (*n* = 20 flowers), bagging mature buds and pollinated with pollen from the same flower; (4) geitonogamy (*n* = 20 flowers), bagging emasculated mature buds and pollinated with pollen from the same plant; and (5) xenogamy (*n* = 20 flowers), bagging emasculated mature buds and pollinated with pollen from different *B. ceiba* trees. Fruit set of open pollination was observed from all the flowers on 17 individuals as control.

**TABLE 2:** Fruit set in *Bombax ceiba* following hand-pollination treatments and natural pollination.

Treatments	No. of trees or flowers	Percentage of fruit set
Bagged only	20 Flowers	0
Emasculation	20 Flowers	0
Self-pollination	20 Flowers	0
Geitonogamy	20 Flowers	0
Xenogamy	20 Flowers	0.85
Open pollination	17 Trees	0.03

### Sample Collection

A total of 378 adult individuals were recorded at this study site, of which 163 adults, including 17 mother trees, were randomly selected for experiments (Figure 2). To accurately assess population size, pollen dispersal distances, and spatial genetic structure (SGS), 2BULU version 6.2.1^3^ was used to obtain the global positioning system (GPS) coordinates of each plant and all the adults were mapped on this study site in 2018 (Figure 2). In late April 2018, two young leaves were collected from all the 163 experimental trees for genotyping. A total of 80 capsules were collected from 17 mother trees in early May 2018. Each tree (family) was composed of 3–7 fruits. In late May 2018, seeds from the same fruit were germinated in a culture dish. At a height of 6 cm, at least 15 seedlings were selected from each fruit/culture dish and a total of 1,190 F1 seedlings were harvested for genotyping.

### Deoxyribonucleic Acid Extraction and Simple Sequence Repeat Genotyping

Total genomic DNA was extracted from leaf tissue by using a modified cetyltrimethylammonium bromide (CTAB) protocol (Doyle, 1990). A total of 13 polymorphic expressed sequence tag (EST)-SSR markers developed by Ju et al. (2015) were applied to all the 1,353 samples (163 adults plus 1,190 seedlings) (Supplementary Table 1). SSR markers are co-dominant, polymorphic, randomly distributed markers in genomes, and are probably selectively neutral (Song et al., 2003). Therefore, SSR markers are widely used in studies of parentage, gene flow, genetic diversity, and population differentiation (Song et al., 2003; Wu et al., 2020).

Polymerase chain reaction amplification of primer pairs was performed with the Veriti 96-Well Thermal Cycler (Applied Biosystems, Foster City, California, United States) by using a 25-μl reaction mixture containing 1 μl of genomic DNA (50 μg/ml stock), 1 μl each of forward and reverse primers (10 μM stock), 12.5 μl of PCR Master Mix, and 9.5 μl of ddH_2_O. Forward primers were labeled with a fluorescent dye [6-carboxy-fluorescein (FAM), carboxytetramethylrhodamine (TAMRA), hexachlorofluorescein (HEX), or 6-carboxy-X-Rhodamine (ROX)]. PCR amplifications were performed as following: an initial denaturation step at 94°C for 5 min, 35 cycles comprising of: (i) denaturation at 94°C for 30 s, (ii) 45 s annealing at temperatures specified below, and (iii) extension at 72°C for 1 min. This was followed by a final extension step at 72°C for 10 min. The annealing temperature was 57°C for BC1, BC3, BC4, BC8, BC9, and BC13 and 60°C for BC2, BC5, BC6, BC7, BC10, BC11, and BC12. To test the utility of the primers, PCR products were detected on 1% agarose gels. Next, the PCR products were resolved on the ABI3730xl Genetic Analyzer (Applied Biosystems, Foster City, California, United States) with an internal lane standards (LIZ) (500) size standard. Fragment data were analyzed by using GeneMarker version 2.4.0 (Softgenetics LLC, State College, Pennsylvania, United States).

### Genetic Diversity

The presence of null alleles was checked by using Micro-Checker version 2.2.3 (Van Oosterhout et al., 2004) and a departure from Hardy–Weinberg equilibrium (HWE) for all the loci was tested by using Genepop version 4.2 (Raymond and Rousset, 1995). For each locus, the genetic diversity for all the sampled adults was characterized by the number of alleles per locus (*N*_*a*_), number of effective alleles (*N*_*e*_), observed heterozygosity (*H*_*o*_), expected heterozygosity (*H*_*e*_), and inbreeding coefficient (*F*_*is*_). The total paternity exclusion probability of the first [Pr(*Ex1*)] and second parent [Pr(*Ex2*)] was also estimated. These analyses were performed by using the Cervus version 3.0.7 program (Marshall et al., 1998).

We tested for signatures of genetic bottlenecks by using two tests available in Bottleneck version 1.3.2.^4^ The Wilcoxon signed-rank test was used, as it has been determined as the most appropriate statistical test with respect to the number of loci employed and the number of sampled individuals per population (Piry et al., 1999). It is available in Bottleneck version 1.3.2 for two mutational models: the stepwise mutation model (SMM) and the two-phase model (TPM) (Di Rienzo et al., 1994). The second test is suitable for evaluating the allele frequency distribution. The alleles were classified into 10 frequency classes, which were then used to test whether the distribution followed the normal L-shaped form.

### Spatial Genetic Structure Analysis

To investigate the SGS of *B. ceiba* adults established within the stand, we conducted spatial autocorrelation analyses based on pairwise kinship coefficients (*F*_*ij*_) between individuals (Loiselle et al., 1995) and against physical distances by using the SPAGeDi program version 1.5d (Hardy and Vekemans, 2002). Twelve distance classes were chosen to achieve the (best) uniform scale over populations as following: 0–200, 200–400, 400–600, 600–800, 800–1,000, 1,000–1,200, 1,200–1,800, 1,800–3,800, 3,800–5,000, 5,000–5,400, 5,400–5,800, and 5,800–6,400 m. The statistical significance of the autocorrelation was tested by 10,000 random permutations with a 95% CI.

### Mating System Analysis

Estimates of mean multilocus (*t*_*m*_) and single locus (*t*_*s*_) outcrossing rates, correlation of *t*_*m*_ within progeny arrays (*r*_*t*_), and multilocus paternity correlation (*r*_*p*_) were calculated with the help of multilocus t and r (MLTR) win 3.4 (Ritland, 2002). The mean number of effective pollen donors (*N*_*ep*_) that participated in cross-pollination was estimated from the within-sibship correlated paternity (*r*_*p*_), which is defined as *N*_*ep*_ = 1/*r*_*p*_. The program was run by using default parameters for the outcrossing rate (*t* = 0.9), parental inbreeding (*F* = 0.1), and paternity correlation (*r*_*p*_ = 0.1). The estimation of mating system indices was made by the expectation-maximization method to ensure convergence; 1,000 bootstraps were used to calculate SE.

### Paternity Analysis

Paternity assignment was conducted by categorical allocation in Cervus version 3.0.7 (Kalinowski et al., 2007). Logarithm of odds (LOD) scores were calculated for all the sampled plants, as every reproductive individual in the population is a potential sire. LOD scores were calculated by determining the likelihood of assignment of a parent relative to the likelihood of arbitrary parents. The simulation parameters were as follows: 10,000 cycles, 380 candidate parents, 0.43 as the proportion of candidate parents sampled, 0.9935 as the proportion of loci typed, and 0.0125 as the rate of typing error (calculated from repeat genotyping). Strict confidence levels were set at 95% and relaxed confidence levels were set at 80%.

Paternity assignment was used to calculate the realized outcrossing rate, multiple paternity per capsule, and realized pollen dispersal distances. Distributions of realized mating and potential mating under random pollinations were tested by using the Kolmogorov–Smirnov test and proportions test for each 100 m increment as well as for the overall study site (Siegel, 1956). Distances between maternal and potential sires represent a theoretical distribution of random mating and were calculated by taking the mean distances between each maternal and all the other plants and then averaging those values for each incremental 100 m distance class (Sokal and Rohlf, 1995). Thus, the proportion of potential mating events reflects the number of maternal/potential sire pairs that occur within each distance class. To standardize the population size and the density, realized pollen dispersal was also plotted against ranked distance between a maternal individual and all the potential sires. Ranked distance was calculated by assigning plant pairs (a value from 0 to 1,300). The resulting distribution was tested for kurtosis (*K*) by using the kurtosis function in R (R Core Team, 2013). Negative kurtosis indicates a flat data distribution (platykurtosis), positive kurtosis indicates a peaked distribution (leptokurtosis), and zero kurtosis indicates a normal distribution. A simple linear regression analysis was applied in R to test whether the proportion of outcross mating declined linearly with ranked distance, following the approach described by Krauss et al. (2009).

## Results

### Bird Observation

At this study sites, *B. ceiba* flowers were pollinated by *Zosterops palpebrosus* (*Z. palpebrosus*), *Pycnonotus xanthorrhous* (*P. xanthorrhous*), *Pycnonotus jocosus* (*P. jocosus*), *Acridotheres cristatellus*, *Sturnus sinensis*, *Lanius* spp., and *Parus* spp. (Table 1). Among them, the visit frequency of *Z. palpebrosus* is the greatest (4.98 ± 0.72 visits per tree per h), while *P. xanthorrhous*, *P. jocosus*, and *Parus* spp. are resident birds (Table 1).

The results of hand-pollination treatments showed that *B. ceiba* fruited solely by xenogamy, with fruit set rate was 85 ± 8.19% (Table 2). For open-pollinated flowers, the number of fruit produced per tree varied from 50 to 300 and the average fruit set rate for 17 trees was 3.27 ± 0.93% (Table 2).

### Genetic Diversity

Null alleles were not detected in any of the 13 microsatellite loci and a low genetic diversity was confirmed. The average number of alleles per locus was 3.62 for adults and 4.15 for F1 seedlings (Supplementary Table 2). The mean *H*_*o*_ and *H*_*e*_ were 0.285 and 0.351 among the adults and 0.343 and 0.389 among the seedlings, respectively (Supplementary Table 2). The overall mean *F*_*is*_ was –0.170 (± 0.074, SE) for the adults and –0.224 (± 0.084, SE) for the offspring, which indicated “heterozygote excess,” but there was no significant difference between them (*P* = 0.638). In addition, *F*_*is*_ in adults and seedlings significantly differed from 0 (*P* = 0.025, *P* = 0.019, respectively). Eight loci in the adults and ten loci in the offspring showed significant deviation from HWE (*P* < 0.05; Supplementary Table 2). Although the calculated genetic diversity measures in the adults were lower than in the seedlings, the differences were not significant for *N*_*a*_ (*P* = 0.335), *N*_*e*_ (*P* = 0.499), *H*_*o*_ (*P* = 0.271), and *H*_*e*_ (*P* = 0.494). Across all the 13 loci, the cumulative expected exclusion probability of the first Pr(*Ex1*) and second Pr(*Ex2*) parents, estimated from adults, was 0.9935 and 0.9999, respectively (Supplementary Table 2).

In the Wilcoxon signed-rank test, the adults (*n* = 163, *P* = 0.305) and the offspring (*n* = 1,190, *P* = 0.641) of *B. ceiba* were found not at mutation-drift equilibrium under the SMM. Under the TPM, the program detected a bottleneck signature for adults (*n* = 163, *P* = 0.034); however, the program did not find this signature for the offspring cohort (*n* = 1,190, *P* = 0.372). However, the allele frequency distribution test showed a mode-shift shape for the adults as well as the offspring of *B. ceiba* in Hainan population (Supplementary Figure 1).

### Spatial Genetic Structure

Spatial genetic structure analysis, by using pairwise differentiation measures, showed significance in the first (0–200 m), second (200–400 m), and eighth distance classes (1,800–3,800 m), with a *F*_*ij*_ value of 0.03615 (*P* = 0.027), 0.03270 (*P* = 0.031), 0.2370 (*P* = 0.033), and 0.01813 (*P* = 0.017), respectively (Supplementary Figure 2). In the seventh (1,200–1,800 m), tenth (5,000–5,400 m), eleventh (5,400–5,800 m), and twelfth distance classes (5,800–6,400 m), *F*_*ij*_ was negative (–0.00394, –0.00077, –0.00238, and –0.00138, respectively), but not significantly different from the expected value (*P* > 0.05).

### Mating System

Multilocus outcrossing rates (*t*_*m*_) for 17 mother plants were equal to 1.000 (Table 3), strongly suggesting that all the seedlings were products of outcrosses. Biparental inbreeding rates (*t*_*m*_-*t*_*s*_) ranged from –0.196 to 0.123, with a mean (± SE) value of –0.063 ± 0.017 among F1 seedlings from 79 capsules (Table 3). The *t*_*m*_-*t*_*s*_ values of the F1 seedlings from capsule R3, R8, R9, R13, R16, and R17 were greater than 0, indicating biparental inbreeding (Table 3).

**TABLE 3:** Mating system, multiple paternity, and outcrossing rate for 17 open-pollinated trees of *Bombax ceiba*.

Maternal plants	Maternal capsules (total capsules)	MLTR estimates			Calculations based on CERVUS results				Proportion outcrossed
		*n*	*t*_*m*_ (SE)	*t*_*m*_-*t*_*s*_ (SE)	*n*		Number of different sires		
					Seeds	Mean seeds/capsule	Mean/capsule	Total/tree	
R1	3 (177)	45	1.000 (0.001)	–0.063 (0.006)	11	3.7	2.00	2	1
R2	5 (236)	75	1.000 (0.001)	–0.113 (0.026)	–	–	–	–	–
R3	5 (295)	75	1.000 (0.001)	0.087 (0.032)	18	3.6	1.50	2	1
R4	5 (119)	75	1.000 (0.001)	0.000 (0.001)	–	–	–	–	–
R5	5 (415)	75	1.000 (0.001)	–0.196 (0.013)	44	8.8	1.75	6	1
R6	3 (191)	45	1.002 (0.001)	–0.168 (0.011)	–	–	–	–	–
R7	5 (116)	75	1.000 (0.001)	–0.028 (0.011)	–	–	–	–	–
R8	6 (223)	95	1.000 (0.001)	0.019 (0.023)	15	2.1	1.00	2	1
R9	3 (196)	45	1.000 (0.001)	0.001 (0.025)	35	11.7	1.67	4	1
R10	3 (310)	45	1.002 (0.001)	–0.025 (0.005)	–	–	–	–	–
R11	5 (203)	75	1.000 (0.001)	–0.012 (0.009)	27	5.4	1.50	3	1
R12	5 (198)	75	1.000 (0.001)	0.000 (0.001)	30	6.0	1.00	3	1
R13	5 (150)	75	1.000 (0.001)	0.004 (0.042)	37	7.4	1.67	6	1
R14	6 (427)	90	1.000 (0.001)	–0.03 (0.017)	43	7.2	1.67	6	1
R15	3 (240)	45	1.000 (0.001)	–0.091 (0.040)	36	12.0	1.67	4	1
R16	6 (326)	90	1.000 (0.001)	0.123 (0.030)	30	5.0	1.00	2	1
R17	6 (386)	90	1.000 (0.001)	0.055 (0.007)	50	8.3	1.50	4	1
Population/combined	79 (4,208)	1,190	1.000 (0.001)	–0.063 (0.017)	376	4.7	1.49	44	1

*t_m_, multilocus outcrossing rate; t_m_ - t_s_, biparental inbreeding estimate.*

*MLTR standard error were calculated from 1,000 bootstraps and are shown in parentheses. Multiple paternity values were calculated on the basis of paternity assignments made in CERVUS.*

Analysis of mating patterns revealed significant correlation in outcrossing rates among siblings (*r*_*t*_ = −0.999 ± 0.000). The estimated correlation of paternity (*r*_*p*_) was 0.265 ± 0.024, indicating that individuals in the family have the opportunity to share the same father (Table 4). The average effective number of pollen donors per maternal plant (*N*_*ep*_) was 3.773. This showed that there was more than one male parent in F1 seedlings derived from a single capsule (Table 4).

**TABLE 4:** Mating system parameters for *Bombax ceiba*.

Parameters	Estimates	SE
Correlation of selfing (*r*_*t*_)	–0.999	0.000
Multilocus correlation of paternity (*r*_*p*_)	0.265	0.024
Average effective number of pollen donors per maternal plant (*N*_*ep*_)	3.773	–
Average pollen dispersal distance (m)	202.89	41.01

*SE calculated from 1,000 bootstrap replicates by resampling maternal families.*

### Paternity Analysis and Multiple Paternity

Paternity assignments revealed the following results: of the 1,190 F1 seedlings, 376 (31.6%) and 605 (50.8%) seedlings were assigned to a single pollen parent in the population at the 95 and 80% confidence levels, respectively. The remaining 585 seedlings (49.2%), which were not assigned to a pollen parent within the study population at < 80% confidence levels, were assumed to have been sired by an individual in the population that had not been sampled or to be a product of immigrant pollen grains from outside Bawangling population.

Paternity analysis revealed high outcrossing rates (1.000) and multiple paternity, as 36 different sires were identified for 376 seeds in 12 families. The mean number of different sires per fruit ranged from 1.0 to 2.0, whereas the number of different sires assigned for paternity per tree ranged from 2 to 6 (Figure 2 and Table 3).

### Pollen Dispersal Distance

**FIGURE 3:**
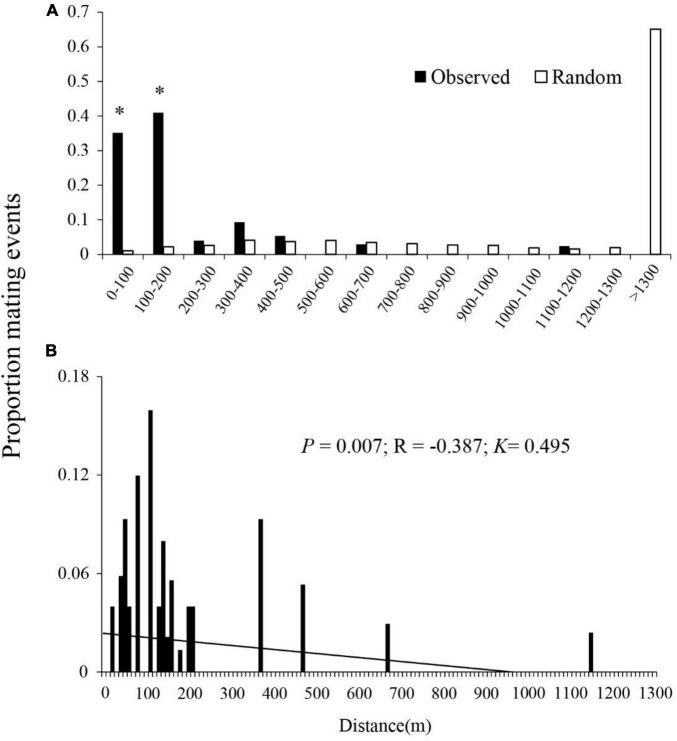
Proportion of realized outcross mating events with distance, showing **(A)** observed and random outcross pollination events with geographic distance (m) between plant pairs and **(B)** proportion of mating events with ranked distance between plant pairs. Realized mating events are based on the results of a paternity assignment of seeds (*n* = 376) collected from 12 *B. ceiba*. Potential mating events are the distances between maternal trees and all the potential sires. *Significant differences (*P* < 0.001) between observed and potential mating events.

The 376 outcrossing F1 seedlings with identified male parents were used to estimate the distance distribution of pollen dispersal. 76.1% of outcrossed pollination events occurred between trees < 200 m apart, 21.5% of outcrossed pollination events occurred between trees 200–700 m apart, and only 2.4% of outcrossed pollination events occurred between trees > 1,100 m apart, respectively (Figure 3). There were significant differences between the observed and the random mating events at 2 of 14 distance classes (all the *P*-values < 0.05), where their proportions (observed/random mating events) were 0.351/0.010 at 0–100 m and 0.410/0.022 at 100–200 m, respectively. Realized mating events were more frequent than events expected for random mating for the plants < 500 m apart, indicating a leptokurtic dispersal at this scale (Figure 3A). The mean realized pollen dispersal distance was 202.89 ± 41.01 m (mean ± SE) (Table 4). The mean distance between maternal plants and all other plants was 1275.59 ± 66.39 m. The maximum realized pollen dispersal distance for paternity assignments, made at the 95% confidence threshold, was 1,140 m (Figure 3B), whereas the distance between maternal plants and their most distant neighbors ranged from 3,172 to 5,958 m.

There was a significant negative correlation (*R* = −0.387, *P* = 0.007) between the frequency of the outcross mating and the distance between mating pairs (Figure 3B). The overall distribution of pollen dispersal distances was leptokurtosis (*K* = 0.495).

## Discussion

Application of molecular markers for the assignment of paternity in *B. ceiba* has produced the same results that expected for our hypothesis. Our findings show that near-neighbor mating is common in generalist-pollinated *B. ceiba* and that the pollen dispersion pattern follows a leptokurtic distribution, which is in accordance with the optimum foraging theory of animals (Pyke, 1984). Our result indicated that *B. ceiba* possesses strict self-incompatibility with limited pollen dispersal distance and low level of paternity, together with low levels of genetic diversity and a significant spatial genetic structure, which may be consequences of near-neighbor mating caused by restricted flight distance of birds and human disturbances.

### Pollen Dispersal Pattern

Our investigation demonstrated limited-distance pollen dispersal in *B. ceiba*, an observation that differs with previous studies suggesting comparatively greater pollen dispersal of flowering plants by bird pollination (Byrne et al., 2007; Bezemer et al., 2016; Krauss et al., 2017). The mean realized pollen dispersal distance of *B. ceiba* plants pollinated with generalist birds was about 200 m, which is significantly higher than that of *Eucalyptus caesia* (*E. caesia*) in a small isolated population (about 80 m) (Bezemer et al., 2016), but significantly lower than that of *Camellia japonica* in a fragmented landscape (about 600 m; Supplementary Table 3; Nakanishi et al., 2020). Pollen dispersal mediated by specialist nectarivorous birds, such as hermit hummingbirds and the word-billed hummingbirds, appears to be farther, as they often move to relatively small, dispersed resources, resulting in the pollen dispersal distance of those plants to be usually greater than 1 km (Byrne et al., 2007; Supplementary Table 3). However, up to 2% of pollination was recorded to occur in the *B. ceiba* population that was up to 1,100 m away, around the Baotie village, where human interference has been increasing and plant density has reduced (Figure 2). Our results are consistent with the research on bird-pollinated plants by Kamm et al. (2009) and Côrtes et al. (2013). Their studies have also shown that human interference and low conspecific plant density may increase pollen dispersal distances (Kamm et al., 2009) because pollinators have to fly further to reach nearby plants. This also reflects that bird pollination can cope with population fragmentation (Byrne et al., 2007), unlike insect pollination (Ren et al., 2017). Further experiments are required, such as landscape genetic analyses, to falsify this.

Our paternity analysis indicates that pollen dispersal follows an extremely leptokurtic distribution within 1,200 m (Figure 3), which was consistent with the optimal foraging theory. In most cases, pollen dispersal is leptokurtic, with the frequency of mating events decreasing with distance between mating pairs (Pyke, 1980; Proctor et al., 1996). Leptokurtic pollen dispersal by animals may be related to their optimum foraging behavior, in which they feed in order to maximize energy reward relative to energy expenditure (Pyke, 1984). Typically, foraging animals travel between flowers on a single plant and then to a nearby neighbor (Harder and Barrett, 1996). Therefore, optimal foraging behavior of nectar- or pollen-feeding animals is likely to result in localized pollen distribution and biparental inbreeding. There was no significant difference between the observed and the random mating events between plants that were 200–500, 600–700, and 1,100–1,200 m apart, indicating that the likelihood of mating was influenced by, but not, strongly dependent on distance. Conversely, there was a significantly higher proportion of observed potential mating events between plants 0–100 and 100–200 m apart, where first-order nearest neighbors and first- to second-order nearest neighbors sired 35 and 41%, respectively, of all the seeds (Figure 3), indicating a frequency of short-distance pollen dispersal in excess of random mating expectations. Therefore, we hypothesize that the high population density of *B. ceiba* and disturbances by human activities such as tourism and farming facilitated short-distance flight of birds, resulting in many near random matings in Baoshan village (Figure 2). In addition, higher correlated paternity was always associated with the nearest-neighbor pollination, which contributes to magnification of the genetic structuring effects (Krauss et al., 2009). This restricted gene flow resulted in high correlated paternity (*r*_*p*_ = 0.265) and may also be the main reason of the SGS of *B. ceiba* at this study site.

### Multiple Paternity

Multiple paternity of *B. ceiba* is observed in this study (Figure 2) and we propose that multiple mating in *B. ceiba* is coupled with a high diversity of sires per maternal tree and facilitated by frequent movement of generalist birds between plants. In flowering plants, multiple paternity is pervasive and occurs when pollen grains from different donors are deposited separately during sequential visits by pollinators or when pollen grains are deposited simultaneously by a single vector that carries pollen grains from several donors (Karron et al., 2006; Llaurens et al., 2008).

The behaviors of generalist birds may contribute to the multiple paternity of *B. ceiba*. In the field, we observed that there are various types of birds that visit *B. ceiba* trees every day, including the large-sized *Acridotheres*, the medium-sized *Pycnonotus* and *Sturnus*, and the small-sized *Zosterops* (Figure 1 and Table 1). They carry pollen grains from various donors to promote multiple paternity. Such results are expected since flowers exposed longer in the pistillate phase of *B. ceiba* (about 4 days) accumulate more pollen on the stigma (Xiang and Ren, 2019). *Zosterops* spp. makes regular visits to various *B. ceiba* trees to drink nectar and is the main floral visitor in our population (Xiang and Ren, 2019; Table 1).

However, multiple paternity in *B. ceiba* (1.49 ± 0.09) was markedly lower than those reported in generalist-pollinated *E. caesia* (4.6 ± 0.3) (Bezemer et al., 2016) and *Banksia hookeriana* (29.8 ± 1.96) (Krauss et al., 2009) in Australia. The lower multiple paternity in *B. ceiba* could be explained by following reasons: (1) Low multiple paternity probably resulted mainly from low fruit setting rate (3.27 ± 0.93%) in *B. ceiba* and restricted flight distance of birds (Table 1), which might cause by human disturbances such as tourism and farming. The blooming *B. ceiba* attracted many tourists and when *B. ceiba* starts blooming, local farmers transplanted rice seedlings in nearby paddies, resulting in decrease in bird visitation frequency and duration; (2) A massive number of flowers on one tree allows birds to meet their nectar needs without moving frequently between trees, which was proved by the restricted pollen dispersal distance (Figure 2 and Figure 3). The high efficiency of pollen removal and low combing behavior of birds (Chen et al., 2019; Song et al., 2019) also made the birds carry a large amount of pollen grains from few pollen donors on their bodies and, thus, have the potential to pollinate multiple ovules in a single visit. One of our observations, where 30 randomly seeds of > 300 seeds of a plant were all from the same father, supported this possibility (Figure 2 and Table 3); and (3) The investigated plant population is not a completely closed population (Figure 2) and some pollen donors might from unlabeled individuals within study population or nearby populations, which might cause underestimation of multiple paternity in our results.

### Obligate Xenogamy and Lower Genetic Diversity

The self-incompatibility in *B. ceiba* is extremely strict and has been documented in the present and previous studies (Khanduri and Kumar, 2017; Table 2). This strict self-incompatibility in *B. ceiba* might be related to flower traits and the behavior of visiting birds. *B. ceiba* flowers are large, erect, and bowl shaped, with numerous stamens surrounding the five-branched stigmas (Figure 1B) and, thus, may easily lead to a massive self-pollen deposition on stigmas during the visit of a generalist bird. Therefore, strict self-incompatibility in *B. ceiba* evolved to ensure outcrossing. This is confirmed by the significant number of aborted fruits observed in the field as well as the withered developing fruits observed in artificial self-pollination treatments.

Our results found a low genetic diversity and obvious SGS in *B. ceiba*. Normally, nectar-feeding birds assist frequent outcrossing and facilitate pollen dispersal over long distances, which are predicted to result in high genetic diversity and weak genetic structure for bird-pollinated plants (Krauss et al., 2017) such as *Banksia attenuata* in Australia (Krauss et al., 2009), *Camellia japonica* in Japan (Nakanishi et al., 2020), and *Penstemon scariosus* (Rodríguez-Peña et al., 2018). In *B. ceiba*, the low genetic diversity and considerable SGS may be an outcome of the genetic bottlenecks (Supplementary Figure 1) caused by human interferences. *B. ceiba* is widely used in engineering, construction, and composite manufacturing, as it is easily available in form of a fast-growing plant species (Saha et al., 2016). A large number of *B. ceiba* trees were cut down for economic development and fruits were collected by local people for silk making to make cloth at the beginning of the twentieth century (Zhang and Zhang, 2018), resulting in significant population decline and low level of population regeneration (Zhao et al., 2016; Hassan, 2018). These human disturbances appear to strongly contribute to the low genetic diversity and bottleneck effects of *B. ceiba*.

## Data Availability Statement

The original contributions presented in the study are included in the article/Supplementary Material, further inquiries can be directed to the corresponding author/s.

## Author Contributions

W-QX assembled and analyzed the data and wrote the initial draft of the manuscript. PM and LT helped with data analyses, reviewed, and revised the draft. M-XR conceived the idea, designed this study, and critically reviewed the manuscript. All the authors contributed to the final version of the manuscript.

## Conflict of Interest

The authors declare that the research was conducted in the absence of any commercial or financial relationships that could be construed as a potential conflict of interest.

## Publisher’s Note

All claims expressed in this article are solely those of the authors and do not necessarily represent those of their affiliated organizations, or those of the publisher, the editors and the reviewers. Any product that may be evaluated in this article, or claim that may be made by its manufacturer, is not guaranteed or endorsed by the publisher.

## Footnotes

1
http://data.cma.cn/

2
http://www.hainanqx.cn

3
https://www.2bulu.com/

4
https://pypi.org/project/Bottleneck/
