# Supplementary material for: Limited-Distance Pollen Dispersal and Low Paternal Diversity in a Bird-Pollinated Self-Incompatible Tree
Source: Front Plant Sci. 2022 Feb 25;13:806217. doi: 10.3389/fpls.2022.806217 (PMC8914170; doi:10.3389/fpls.2022.806217)
Supplement: Supplementary file 1 [file Data_Sheet_1.docx]

**SUPPLEMENTARY MATERIAL**


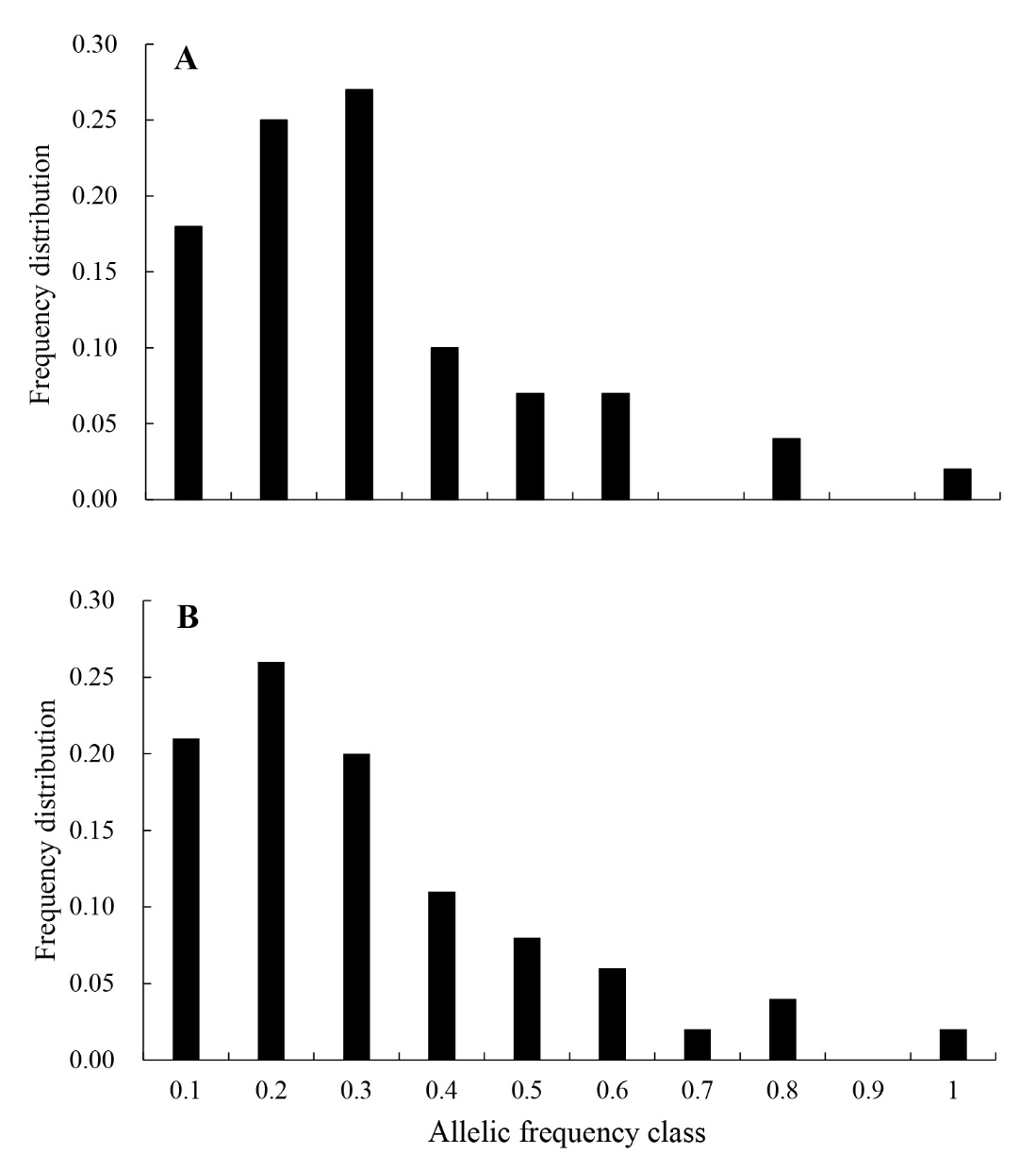


**Supplementary Figure 1.** Mode-shift test for bottleneck analysis of adult **(A)** and offspring **(B)** of *Bombax ceiba* in Hainan population.


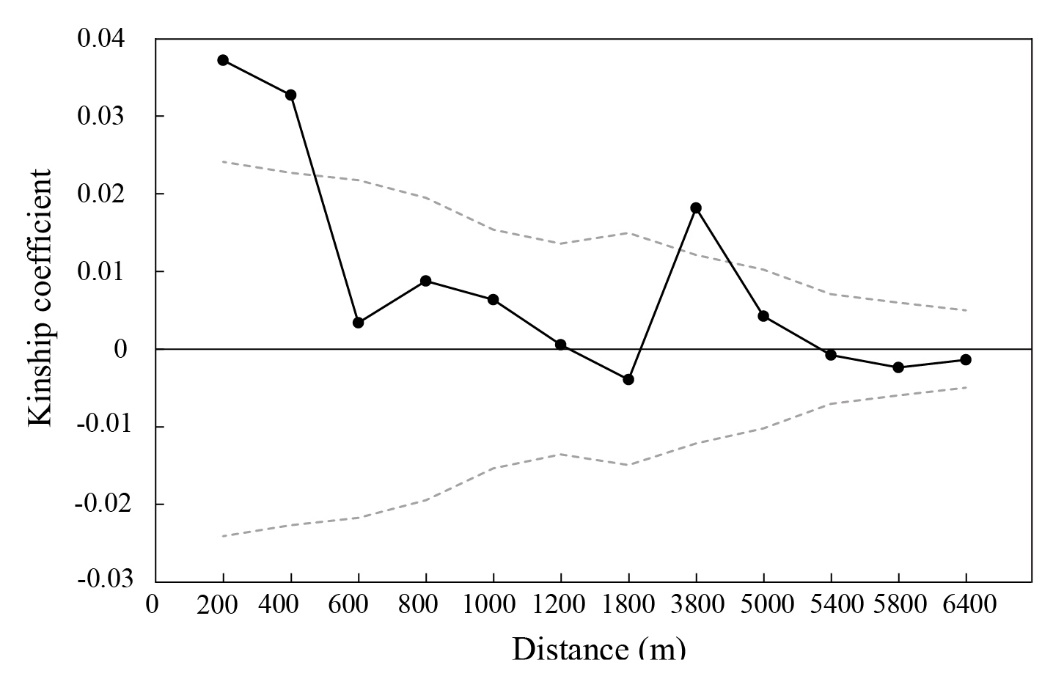


**Supplementary Figure 2.** Graph of kinship coefficients in twelve distance classes among the adults within the *Bombax ceiba* study stand. The dashed lines represent the upper and the lower 95% confidence limits.

| **Supplementary Table** **1. Characterization and annealing temperatures (*T*_a_) of 13 polymorphic EST-SSR markers of *Bombax ceiba* developed by Ju et al. (2015)** | | | | | |
| --- | --- | --- | --- | --- | --- |
| Locus | Motif | Allele range (bp) | *T*_a_ (°C) | Lables | GenBank accession no. |
| BC1 | (CTT)_7_ | 270–273 | 57 | 5'-FAM | KP216639 |
| BC2 | (TA)_11_ | 250–268 | 60 | 5'-HEX | KP216640 |
| BC3 | (TTC)_7_ | 204–207 | 57 | 5'-ROX | KP216642 |
| BC4 | (TCA)_7_ | 153–156 | 57 | 5'-TAMRA | KP216643 |
| BC5 | (CAG)_7_ | 128–134 | 60 | 5'-FAM | KP216644 |
| BC6 | (TG)_10_ | 149–165 | 60 | 5'-HEX | KP216645 |
| BC7 | (CA)_10_ | 248–250 | 60 | 5'-ROX | KP216646 |
| BC8 | (CGA)_8_ | 156–168 | 57 | 5'-TAMRA | KP216647 |
| BC9 | (GACT)_6_ | 134–138 | 57 | 5'-FAM | KP216648 |
| BC10 | (ACA)_8_ | 213–216 | 60 | 5'-HEX | KP216649 |
| BC11 | (CAGC)_6_ | 316–320 | 60 | 5'-ROX | KP216650 |
| BC13 | (CTG)_7_ | 270–281 | 57 | 5'-TAMRA | KP216652 |

| **Supplementary Table 2. Genetic diversity across 13 microsatellite loci within all adults (n=163) and offspring (n=1190) of *Bombax ceiba*.** | | | | | | | | | | | | | |
| --- | --- | --- | --- | --- | --- | --- | --- | --- | --- | --- | --- | --- | --- |
| Locus | Adults (n = 163) | | | | | | |  | Offspring (n = 1190) | | | | |
|  | *N_a_* | *N_e_* | *H_o_* | *H_e_* | *F_is_* | Pr (*Ex1*) | Pr (*Ex2*) |  | *N_a_* | *N_e_* | *H_o_* | *H_e_* | *F_is_* |
| BC1 | 2 | 1.416 | 0.281 | 0.207 | -0.541^***^ | 0.473 | 0.356 |  | 6 | 2.823 | 0.473 | 0.485 | -0.035^ns^ |
| BC2 | 4 | 2.019 | 0.151 | 0.275 | -0.041^ns^ | 0.628 | 0.472 |  | 2 | 1.652 | 0.129 | 0.236 | 0.047^ns^ |
| BC3 | 3 | 1.605 | 0.242 | 0.326 | -0.093^*^ | 0.625 | 0.511 |  | 3 | 1.725 | 0.296 | 0.375 | -0.106^*^ |
| BC4 | 3 | 2.216 | 0.323 | 0.349 | 0.026 ^ns^ | 0.634 | 0.43 |  | 4 | 2.71 | 0.602 | 0.631 | 0.106^***^ |
| BC5 | 6 | 1.779 | 0.235 | 0.353 | -0.653^***^ | 0.534 | 0.396 |  | 5 | 2.665 | 0.271 | 0.226 | 0.062^*^ |
| BC6 | 4 | 2.203 | 0.332 | 0.337 | -0.013^ns^ | 0.787 | 0.621 |  | 2 | 1.377 | 0.334 | 0.392 | -0.730^***^ |
| BC7 | 2 | 1.709 | 0.253 | 0.164 | 0.051 ^**^ | 0.633 | 0.503 |  | 4 | 1.095 | 0.339 | 0.417 | -0.061^ns^ |
| BC8 | 3 | 1.674 | 0.603 | 0.58 | -0.116^*^ | 0.472 | 0.328 |  | 5 | 2.562 | 0.581 | 0.695 | -0.460^**^ |
| BC9 | 3 | 1.356 | 0.273 | 0.347 | -0.041^ns^ | 0.879 | 0.712 |  | 4 | 2.538 | 0.338 | 0.385 | -0.363^**^ |
| BC10 | 6 | 3.058 | 0.232 | 0.39 | 0.045^*^ | 0.512 | 0.397 |  | 7 | 2.714 | 0.268 | 0.321 | -0.728^***^ |
| BC11 | 3 | 2.237 | 0.145 | 0.245 | -0.207^**^ | 0.469 | 0.295 |  | 4 | 2.319 | 0.149 | 0.207 | -0.331^**^ |
| BC12 | 3 | 1.63 | 0.401 | 0.571 | -0.664^***^ | 0.432 | 0.265 |  | 3 | 1.683 | 0.348 | 0.472 | 0.128 ^***^ |
| BC13 | 5 | 2.902 | 0.239 | 0.425 | 0.036 ^ns^ | 0.537 | 0.406 |  | 5 | 1.894 | 0.327 | 0.221 | -0.436^***^ |
| Mean | 3.62 | 1.985 | 0.285 | 0.351 | -0.170^***^ |  |  |  | 4.15 | 2.135 | 0.343 | 0.389 | -0.224^***^ |
| Total | 47 |  |  |  |  | 0.9935 | 0.9999 |  | 54 |  |  |  |  |
| *N_a_*, no. of observed alleles; *N_e_*, no. of effective alleles; *H_o_* and *H_e_*, observed and expected heterozygosity, respectively; *F_is_*, inbreeding coefficient; Pr (*Ex1*) and Pr (*Ex2*), paternity exclusion probability of the first and second patent, respectively. High significant departure from Hardy-Weinberg equilibrium with heterozygote excess is indicated as ****P* < 0.001, ***P* < 0.01, **P* < 0.05 and ns (not significant). | | | | | | | | | | | | | |

| **Supplementary Table 3. Comparisons of bird pollinations in different continents.** | | | | | |  |
| --- | --- | --- | --- | --- | --- | --- |
| Ranges | Plant species | Pollinators | Pollen dispersal distance (m) | Outcrossing rate | References | |
| Africa | *Salvia africana-lutea* | Sunbirds/*Zosterops pallidus*/Honeyeater | No data | 0.38-0.81 | Wester and Claßen-Bockhoff, 2006 | |
|  | *Protea caffra* | Sunbirds | No data | 0.59 | Steenhuisen et al., 2012 | |
|  | *Babiana ringens* | Sunbirds | No data | 0.32 | de Waal et al., 2012 | |
| South America | *Justicia secunda* | Hummingbird | 100 | No data | Linhart and Feinsinger, 1980 | |
|  | *Delphinium nuttallianum* | Hummingbird | 400 | No data | Schulke and Waser, 2001 | |
|  | *Heliconia pogonantha* | Hummingbird | >1000 | 1.00 | Stiles, 1975 | |
|  | *Ipomopsis aggregat* | Hummingbird | >1000 | 0.66 | Campbell, 1998 | |
| Australia | *Eucalyptus caesia* | Honeyeater | 80 | 0.72 | Bezemer et al., 2016 | |
|  | *Banksia hookeriana* | Honeyeater | 29.9 | 0.81 | Krauss et al., 2009 | |
|  | *Banksia sphaerocarpa* var. *caesia* | Honeyeater | 8.3-84.4 | 0.91 | Llorens et al., 2012 | |
|  | *Calothamnus quadrifidus* | Honeyeater | 30-5000 | 0.05-0.82 | Byrne et al., 2007 | |
| Asia | *Bruguiera gymnorrhiza* | sunbirds /honeyeaters | 7.3-98.9 | 0.86 | Wee et al., 2015 | |
|  | *Camellia japonica* | *Zosterops japonica* | 0-1000 | 0.96 | Nakanishi et al., 2020 | |
|  | *Bombax ceiba* | *Zosterops palpebrosa / Pycnonotus jocosus / Brachypodius atriceps* | 15-1140 | 1.00 |  | |
